# Harnessing Current Knowledge of DNA N6-Methyladenosine From Model Plants for Non-model Crops

**DOI:** 10.3389/fgene.2021.668317

**Published:** 2021-04-29

**Authors:** Sadaruddin Chachar, Jingrong Liu, Pingxian Zhang, Adeel Riaz, Changfei Guan, Shuyuan Liu

**Affiliations:** ^1^State Key Laboratory of Crop Stress Biology for Arid Areas, College of Horticulture, Northwest A&F University, Yangling, China; ^2^Department of Biotechnology, Faculty of Crop Production, Sindh Agriculture University, Tandojam, Pakistan; ^3^College of Mathematics and Statistics, Northwest Normal University, Lanzhou, China; ^4^Biotechnology Research Institute, Chinese Academy of Agricultural Sciences, Beijing, China; ^5^Deaprtment of Biochemistry, Faculty of Life Sciences, University of Okara, Okara, Pakistan

**Keywords:** epigenetic modification, DNA methylation, N6-methyladenosine, gene expression, artificial intelligence, deep learning

## Abstract

Epigenetic modifications alter the gene activity and function by causing change in the chromosomal architecture through DNA methylation/demethylation, or histone modifications without causing any change in DNA sequence. In plants, DNA cytosine methylation (5mC) is vital for various pathways such as, gene regulation, transposon suppression, DNA repair, replication, transcription, and recombination. Thanks to recent advances in high throughput sequencing (HTS) technologies for epigenomic “Big Data” generation, accumulated studies have revealed the occurrence of another novel DNA methylation mark, N6-methyladenosine (6mA), which is highly present on gene bodies mainly activates gene expression in model plants such as eudicot Arabidopsis (*Arabidopsis thaliana*) and monocot rice (*Oryza sativa*). However, in non-model crops, the occurrence and importance of 6mA remains largely less known, with only limited reports in few species, such as *Rosaceae* (wild strawberry), and soybean (*Glycine max*). Given the aforementioned vital roles of 6mA in plants, hereinafter, we summarize the latest advances of DNA 6mA modification, and investigate the historical, known and vital functions of 6mA in plants. We also consider advanced artificial-intelligence biotechnologies that improve extraction and prediction of 6mA concepts. In this Review, we discuss the potential challenges that may hinder exploitation of 6mA, and give future goals of 6mA from model plants to non-model crops.

## Introduction

Hereditary changes in activity and function of a gene caused by the direct alteration of DNA sequence like deletions, insertions, point mutations and translocations is known as Genetics. While epigenetics is the phenomenon of heritable changes in the activity or function of a gene without causing any change in DNA sequence itself (Heard and Martienssen, 2014; Allis and Jenuwein, 2016). The epigenetic mechanism mainly including histone posttranscriptional modifications (PTMs) and DNA methylation plays a vital role in gene expression and various biological functions (Heard and Martienssen, 2014; Allis and Jenuwein, 2016). In consideration of epigenetic enzymatic systems, for dynamically installing, removing, and epigenetic modifications; epigenetic regulation is installed by “writers,” removed by “erasers,” and read by “readers” with a battery of proteins to facilitate biochemical modifications on DNA and histone proteins (Mozgova and Hennig, 2015; Allis and Jenuwein, 2016). Recently, it has enabled the advancement in breeding approaches providing new source of variability originating from epi-alleles (Gallusci et al., 2017; Springer and Schmitz, 2017).

DNA methylation is another most commonly studied epigenetic mark that is responsible for regulating numerous cellular pathways along with gene expression (Feng et al., 2010; Smith and Meissner, 2013). From the genomic DNA of diverse species, numerous methylated bases have been reported like 5-methylcytocine (5mC), N4-methylcytocine (4mC), and N6-methyladenine (6mA). As a well-studied repressive mark, 5mC has shown dynamic regulation of establishing, maintaining, and active removal of their activities, and also involved in gene silencing, transposon insertions, deletion, genome stability and gene expression regulation during all progressive stages in plants (Niederhuth and Schmitz, 2017; Gallego-Bartolomé et al., 2018). Recent reports have revealed that DNA methylation performs a significant role in controlling the growth, development and ripening in horticultural plants; During plant growth, 5mC levels show tissue specific features while during development the methylation levels exhibits variations (Lang et al., 2017; Cheng et al., 2018; Huang et al., 2019; Sabbione et al., 2019; Liang et al., 2020; Tang et al., 2020).

As DNA 5mC and other histone modifications have previously been discussed in other reviews in non-model crops (Duan et al., 2018; Tang et al., 2020), now we merely focus on the comparatively uncharacterized DNA 6mA methylation in plants. In this Review, we introduce the dynamic distribution pattern and motifs of 6mA in plants. Recent advances in high-throughput sequencing (HTS) technologies for detecting 6mA, we have also summarized the 6mA methyltransferases (writers) and demethylases (erasers), the molecular and biological functions of 6mA in plants. We also consider advanced artificial-intelligence biotechnologies that improve extraction and prediction of 6mA concepts. Finally, we discuss the potential challenges that may hinder exploitation of 6mA, and in the end we have given future perspectives of 6mA from model plants to non-model crops.

## 6mA Detection Methods in Plants

**FIGURE 1:**
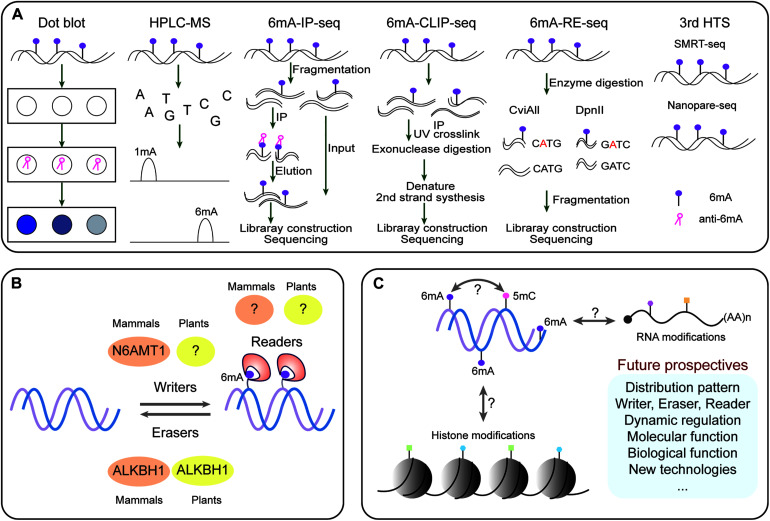
The dynamic DNA 6mA modification in plants. **(A)** Methods for detecting 6mA. **(B)** The enzymatic systems of 6mA. **(C)** Potential association with other epigenetic modifications and future perspective of 6mA. In plants, 6mA can be detected by various methods, such as Dot blot, HPLC–MS, 6mA-IP-seq, 6mA-CLIP-Exo-seq, 6mA-RE-seq, SMRT-seq, and Nanopore-seq. The first two can only detect global 6mA with both qualitative and quantitative analyses but not detect specific 6mA sites; 6mA-IP-seq, 6mA-CLIP-Exo-seq, and 6mA-RE-seq can decipher genome-wide 6mA sites or peaks at a large scale. While, SMRT-seq and Nanopore-seq can identify single-base resolution 6mA and illustrate computational motifs and distribution patterns for the accuracy and robustness of 6mA detection. Currently, we are only known that N6AMT1 is a 6mA writer in mammals, ALKBH1 acts as both erasers in mammals and plants; it remains unknown the readers for recognizing 6mA site on the genome.

Detection of DNA methylation has evolved over the years to become progressively accurate and sensitive. In plants, various methods have been developed for 6mA detection, such as Dot blot, high-performance liquid chromatography combined with mass spectrometry (HPLC-MS/MS), methylated DNA immunoprecipitation sequencing (MeDIP-seq) for 6mA (6mA-IP-seq), 6mA-IP-seq combining photo-crosslinking with exonuclease digestion (6mA-CLIP-Exo-seq), and methylated DNA with restriction enzyme digestion followed by sequencing (6mA-RE-seq), and third-generation high-throughput sequencing (HTS) technologies including single-molecule real time sequencing (SMRT-seq) and Nanopore sequencing (Nanopore-seq) (Figure 1A; Liang et al., 2020).

HPLC-MS/MS has been used for detecting the lowest levels of up to 0.00001% 6mA in the genome of different organisms (Huang et al., 2015). An alternative approach, dot blot assay with specific 6mA antibodies was applied, but with limitation of detection resolution (Liang et al., 2020). These two detection methods have been successfully used for the presence of 6mA in certain organisms, but they are unable to provide genomic location of 6mA.

For determining the genome wide 6mA sites, numerous methylation sensitive sequencing approaches have been established. Firstly, 6mA-IP-seq and its related 6mA-CLIP-Exo-seq can achieve near single-nucleotide resolution of 6mA (Chen et al., 2015). However, 6mA-IP-seq has also limitations; it is antibody dependent precisely recognizing 6mA. For example, the antibody used to detect 6mA can also detect m^6^A in eukaryotic mRNAs (Luo et al., 2014); thus these antibody approaches are not quantitative and maybe confounded by recognition of other adenine modifications (such as m^6^A and m^1^A in RNA), and require pure DNA with RNase enzyme digestion.

Another method for detecting 6mA-RE-seq involves radioactive methylation of DNA coupled with restriction digestion, electrophoresis and sequencing (Fu et al., 2015; Liang et al., 2020). Digestion of methylated DNA with the restriction enzymes such as *Dpn*I (recognize 5′-G6mATC-3′), *Dpn*II (recognize unmethylated 5′-GATC-3′), and *Cvi*AII (recognize unmethylated 5′-CATG-3′) can accurately detect and identify 6mA sites at single-base resolution (Liang et al., 2020). But 6mA-RE-seq is limited in detection of specific sequence motifs with relative low proportions of 6mA in the whole genome.

Single-molecule real time sequencing (SMRT-seq) is a third generation sequencing technique, it offers accurate sequence reads and measures the kinetic rate of nucleotide incorporation during sequencing (Flusberg et al., 2010). SMRT sequencing has been used to map 6mA and 5mC simultaneously in *Escherichia coli* (Fang et al., 2012) 6mA in *Caenorhabditis elegans* (Greer et al., 2015) and 6mA in green algae and human lymphoblastoid cells (hLCLs) (Zhu et al., 2018). SMRT can detect every DNA modification at single base resolution (Zhu et al., 2018). Recently, 6mA has been detected on genome-wide level in eudicot model *Arabidopsis thaliana* (Liang et al., 2018b) and monocot model rice (*Oryza sativa*) (Zhang et al., 2018; Zhou et al., 2018), as well as in non-model plants *Casuarina equisetifolia* (Ye et al., 2019), woodland strawberry (*Fragaria vesca*) (Xie et al., 2020), and soyabeans (*Glycine max)* (Yuan et al., 2020), and fig (*Ficus carica*) (Usai et al., 2021). While combined SMRT sequencing and 6mA-IP-Seq have been used to detect genome wide 6mA levels in *Arabidopsis* and rice (Liang et al., 2018b; Zhang et al., 2018; Zhou et al., 2018), and they reveals more efficient information for dynamic distribution and pattern of 6mA compared to these 6mA studies in non-model plants. In addition, *in silico* database for storing genome-wide 6mA information have also been developed for integrative 6mA and other DNA modifications in both rice and Rosaceae from SMRT sequencing datasets (Liu Z. Y. et al., 2019; Zhang et al., 2020).

For the genomes having lower DNA abundance modifications, the sensitivity and specificity of detection methods need to be balanced. High sensitivity but low specificity may yield false positive results, while weak signals may be missed with high specificity but low sensitivity. To achieve relatively high sensitivity and high specificity, multi-strategy cross-validation is essential in order to detect a rare DNA modification. A combination of complimentary techniques is ideal for convincingly detecting rare modifications such as 6mA, as each technique has its own limits.

## DNA 6mA Methyltransferases and Demethylases in Plants

**FIGURE 2:**
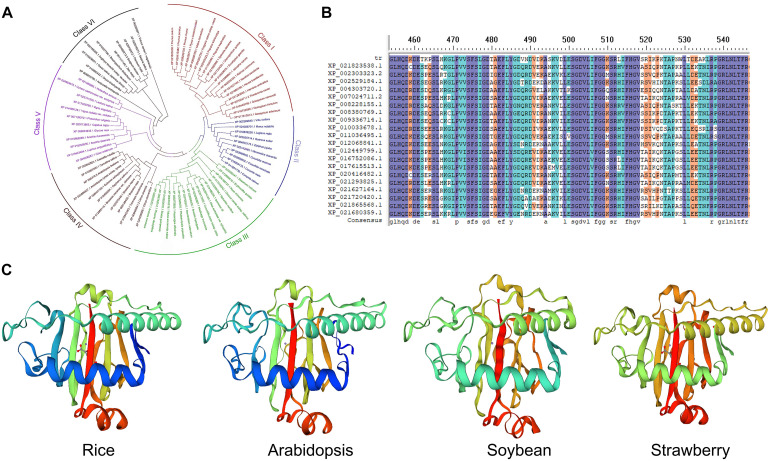
The ALKBH1 orthologs in plants. **(A)** NJ tree and **(B)** sequence alignment of ALKBH1 orthologs in plants. **(C)** Protein structure of ALKBH1 orthologs in rice, *Arabidopsis*, soybean, and strawberry. Sequences of selected ALKBH1 putative orthologs were downloaded from the NCBI database, and then aligned. Phylogenetic tree was established by MEGA7 using neighbor-joining (NJ) tree with 1,000 replicate bootstrap support. Homology-modeling-based structural prediction of the ALKBH1 orthologs followed by the rice ALKBH1 structure (5XEG; Zhou et al., 2018).

In DNA the formation of 6mA takes place by recognition of respective adenine by specific enzymatic systems, including methyltransferases (writers), demethylases (erasers) and DNA 6mA methylation binding proteins (readers). In mammals the AlkB family proteins have also been found to act as 6mA demethylase (Figure 1B; Liang et al., 2020). Recently the rice homologous gene *OsALKBH1* has been identified (Zhou et al., 2018). For further confirming the function of OsALKBH1 in demethylation two mutant lines were obtained by Clustered Regularly Interspaced Short Palindromic Repeats (CRISPR)-associated protein 9 (Cas9). No visible variation was observed in phenotype of these lines except earlier heading displayed two to threefold increase in 6mA levels. While, knockout of *OsALKBH1* did not display any effect on total 5mC levels, suggesting that overall 5mC levels remained unaffected with the increase in 6mA levels in rice genome (Zhou et al., 2018). The OsALKBH1 protein has been shown to be localized in the cytoplasm and nucleus (Zhou et al., 2018), and the putative orthologs of ALKBH1 proteins may be well conserved across plant kingdoms (Figures 2A,B). Remarkably, protein structure modeling shows that ALKBH1 putative orthologs are well conserved in rice, *Arabidopsis*, soybean and strawberry (Figure 2C). Further observation along with its demethylase activity on ssDNA indicates that OsALKBH1 may carryout 6mA demethylation from both RNA and DNA (Zhou et al., 2018), much like the condition in which the similar enzymes are involved methylating both 6mA in DNA and m^6^A in RNA (O’Brown and Greer, 2016).

Recently, N6AMT1 representing a distinct MT-A70 methyltransferase was characterized in mammals (Xiao et al., 2018). However, although it has had orthologs of MT-A70 family proteins in plant kingdoms, it is unknown whether these proteins can act as 6mA methyltransferases in plants (Liang et al., 2020). In addition, we are also still unknown about 6mA readers in both mammals and plants. So far only OsALKBH1 in rice has been revealed as a potential 6mA eraser, it would be interesting to identify and characterize other 6mA writers, erasers and even readers in both model plants and non-model crops that are responsible for altering the genomic DNA.

## Molecular Function of 6mA in Plants

**FIGURE 3:**
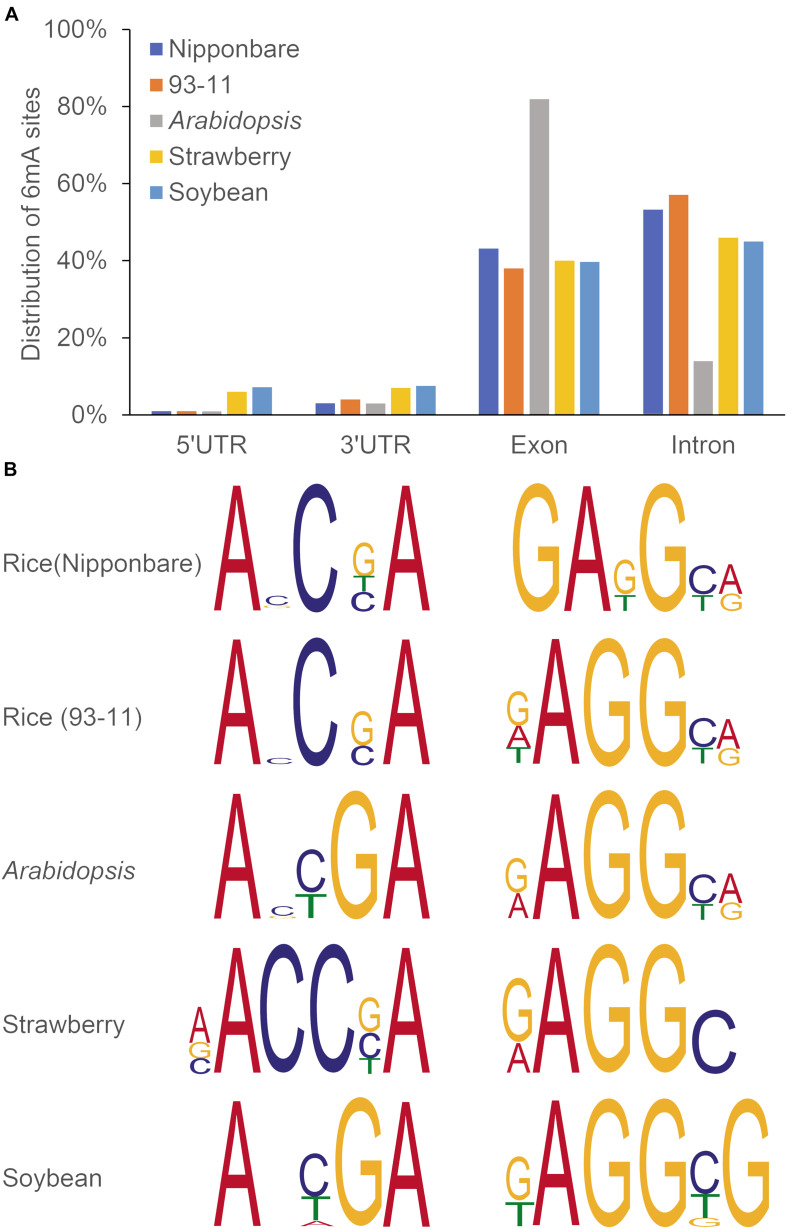
Distribution pattern **(A)** and representative motifs **(B)** of 6mA in in rice, *Arabidopsis*, soybean, and strawberry. Gene bodies include exons, introns, and 5′ and 3′ UTRs. Data were reproduced by previous studies (Liang et al., 2018b; Zhang et al., 2018; Xie et al., 2020; Yuan et al., 2020).

Recently several studies have confirmed the genome-wide distribution of 6mA in plants. Thanks to advance in 6mA HTS methods as mentioned above, it allows to exam whether 6mA could influence transcription by altering transcription factor binding or modifying chromatin structure or not. For example, Liang et al. measured the genome-wide distribution and levels of 6mA in gDNA of *Arabidopsis*, applying different approaches, including DNA dot blot, using a specific 6mA antibody, SMRT sequencing and LC-MS/MS, which could detect the strand specific 6mA sites at single nucleotide resolution (Liang et al., 2018b). In *Arabidopsis* genome, 6mA is present in significant levels and broadly distributed with dynamic variation in its levels at different developmental stages in different tissues, which around 30% of 6mA exist on gene bodies (Liang et al., 2018b). Detected by SMRT-seq, a relatively abundant 6mA sites were located on gene bodies in other plants (Zhang et al., 2018; Xie et al., 2020; Yuan et al., 2020). Within the gene bodies, about half of 6mA sites were located in exons (more than 80% in exons for *Arabidopsis*) (Figure 3A). Of important note, plants may share consensus sequence elements (motifs), when compared in *Arabidopsis*, rice, soybean and strawberry by a search for 6mA consensus motifs (Figure 3B). These results may indicate conserved and essential roles of 6mA in regulation of gene expression across plant species.

Remarkably, in plants and mammals, 6mA has been shown to have opposite effects on the transcription factor binding. The distribution and function of 6mA is found to be conserved in most of the mammalian species, 6mA is found to be enriched around TSS and then actively transcribed in gene expression (Liang et al., 2020). Similar kind of phenomenon was also observed in *Arabidopsis* (Liang et al., 2018b) and later in rice (Zhang et al., 2018). Comparison 6mA methylome and RNA sequencing data in plants showed that 6mA is positively correlated with actively transcribed genes (Liang et al., 2018a,b). Thus, it seems that 6mA is a general mark of active genes, though it is presently not clear whether it plays any role in the dynamic gene expression regulation. While Zhou et al. (2018) reported the 6mA methylome in rice by performing 6mA immunoprecipitation followed by sequencing, which revealed that 6mA peaks are evenly distributed in the chromosomes and correlate with 5mC distribution. Contrastingly, the well-known 5mC is correlated with transcription repression. In addition, 6mA has been shown to associate with accessible chromatin regions, 3D chromatin structures and other types of epigenetic modifications (e.g., histone modifications, non-coding RNAs) to regulate gene transcription in plants (Liang et al., 2021; Zhou et al., 2021). Thus, it needs more investigation on what molecular role of 6mA has during transcription in plants.

## Biological Function of 6mA in Plants

6mA has been found to be positively associated with expression of key genes, which may be responsible for discrepancy in both developmental cues and stress tolerance in plants. In rice, mutations in *DDM1 (Deficient in DNA Methylation 1)* lead to decreased 6mA levels and defects in plant growth (Zhang et al., 2018). By CRISPR/Cas9 gene editing technology, double mutant *ddm1a/1b* show dwarf, decreased seed-setting rate and infertility phenotype compared with wild-type plants (Zhang et al., 2018). To answer the question how *DDM1* regulates rice growth, this study further identified 6mA modified genes that were differentially expressed in *ddm1a/1b*, such as *Grain number, plant height, and heading date7 (GHD7)*, *BR-Deficient Dwarf1 (BRD1)*, and *DWARF7 (DWF7)* (Zhang et al., 2018). Similarly, mutation in *OsALKBH1* exhibits earlier heading date with two to threefold increases of 6mA levels (Zhou et al., 2018). And also, *in Arabidopsis*, flowering promoters *FLOWERING LOCUS T (FT)* and *FRUITFULL (FUL)* were associated with new 6mA modification, when increased their expression during floral transition (Liang et al., 2018b). Together, these examples likely imply that association between 6mA modification and gene transcription may contribute to plant developmental cues.

DNA methylation control a wide range of (a)biotic stress conditions. Several studies have revealed positive correlations of DNA methylation and key genes for stress responses in plants (Popova et al., 2013; Migicovsky et al., 2014; Zheng et al., 2017). Study on 6mA modification among rice cultivars from *japonica* and *indica* group revealed varied response under different environmental conditions (Zhang et al., 2018). For example, *japonica* cultivar (Nippobare) was found to be more tolerant to cold and salt stress while *indica* (93-11) more tolerant to heat stress due to difference in 6mA levels. Thus, essential roles of 6mA associated with long-term environmental adaptations can be exploited in further plant improvement programs.

Hence, 6mA is yet an unknown DNA epigenetic modification linked to actively expressed genes in model plants *Arabidopsis* and rice and might play an active role in plant development and stress responses. It will be important for future studies to reveal how 6mA regulates gene expression, identification and characterization of interacting proteins will help in understanding the functions and mechanisms by which 6mA influences gene expression in plants, especially for non-model plants.

## 6mA Predictors by Machine Learning and Deep Learning

Although several experimental methods have been suggested for the genome-wide identification of 6mA sites, these approaches are time consuming and laborious. Given that genome-wide 6mA profiles have developed with single-base resolution (Liang et al., 2020), and since the distribution of the 6mA sites in the genome is non-random with consensus GAGG motif resent in *Arabidopsis*, rice and other eukaryotic organisms that is only partially conserved (Liang et al., 2018a). A precise and reliable method for identification of 6mA sites is *in silico* machine learning and deep learning invaluable to gain a better understanding of the regulatory mechanism of 6mA. Previous studies have demonstrated that deep learning is a powerful approach for sequence analysis and classification in bioinformatics (Chen et al., 2019; Lv et al., 2019; Yu and Dai, 2019). In order to identify genome wide 6mA sites, several machine learning methods have recently been developed (Table 1; Feng et al., 2019; Yu and Dai, 2019; Lv et al., 2019, 2020; Pian et al., 2020; Wang et al., 2020). For example, Chen et al. recently presented a 6mA benchmark dataset comprising 880 6mA sites and 880 non-6mA sites in the genome of rice (Chen et al., 2019), which was signified as 6mA-rice-Chen. They constructed a support vector machine (SVM) based tool called i6mA-Pred for identifying 6mA sites in the rice genome by using several manually designed DNA sequence features. It was documented that on the rice genome dataset, i6mAPred had an accuracy of ∼83% (Chen et al., 2019). In another study, Pian et al. (2020) suggested a method based on the markov model (MM) for prediction of 6mA sites, called MM-6mAPred. Based on the 6mA-rice-Chen benchmark dataset, they developed and evaluated their MM-6mAPred. MM-6mAPred has been reported to outperform i6mA-Pred in the 6mA sites prediction with the sensitivity and specificity of ∼89 and ∼90%, respectively, having the overall accuracy of ∼90% (Pian et al., 2020). For the identification of 6mA in the rice genome, Tahir et al. recommended another computational method, called iDNA6mA (Tahir et al., 2019). They also trained and evaluated their iDNA6mA on the 6mA-rice-Chen dataset, and they discovered that in prediction performance, iDNA6mA outperformed i6mA-Pred. A method, called SDM6A, was proposed by Basith et al. to predict 6mA sites in the rice genome (Basith et al., 2019). SDM6A is an ensemble technique that uses many methods of encoding features and classifiers for machine learning. They trained and assessed their SDM6A on the basis of the 6mA-rice-Chen benchmark dataset, and they discovered that on the 6mA-rice-Chen benchmark dataset, SDM6A outperformed i6mA-Pred and iDNA6mA.

**TABLE 1:** Summarizing recent machine learning based 6mA prediction approaches.

Method name	Description	Specie	References
i6mA-Pred	The support vector machine approach (SVM) to identify 6mA sites in rice genome with 83% accuracy, in which the DNA sequences are effectively formulated and encoded through the use of chemical property and nucleotide frequency dependent on the SVM approach	Rice genome	Chen et al., 2019
SNNRice6mA	A simple and lightweight deep learning model approach for identifying 6mA from rice genome, its evaluation is based on five metrics such as sensitivity, accuracy, specificity, area under the curve (AUC) and Matthews correlation coefficient (MCC)	Rice genome	Yu and Dai, 2019
i6mA-DCNP	A high-quality computational method to identify and predict 6mA sites in the rice genome. This prediction approach is based on encoding the genomic DNA samples using dinucleotides composition and the optimized dinucleotide-based DNA properties	Rice genome	Kong and Zhang, 2019
Sequence-based DNA N6-methyladenine predictor (SDM6A)	A sequence-based two-layer method for effectively predicting novel putative 6mA sites and non-6mA sites in the rice genome	Rice genome	Basith et al., 2019
iDNA-MS	Utilization of random forest for identifying 6mA, 5hmC, and 4mC sites in multiple species	Multiple species, *Fragaria vesca*, *Arabidopsis thaliana*, *Rosa chinensis, Casuarina equisetifolia*	Lv et al., 2020
Meta-i6mA	An interspecies prediction tool to identify DNA 6mA sites of plant genome through the use of informative features in an integrative machine learning framework	Rice genome	Hasan et al., 2020
csDMA	A method for identifying and predicting 6mA in various species through Chou’s 5-step rule using three encoding features and different algorithms to produce the feature matrix	Multiple species	Liu Z. et al., 2019
iDNA6mA- Rice	The machine learning random forest algorithm to formulate the sample as an input to differentiate between the methylated and non-methylated sites in rice genome for evaluating 6mA sites	Rice genome	Lv et al., 2019
iDNA6mA-PseKNC	A sequence-based prediction approach that allows 100% accuracy and 96% precision to identify DNA 6mA sites without using complicated mathematical formulas	Multiple species	Feng et al., 2019
iDNA 6mA	A deep learning method, based on the conventional neural network for identifying 6mA sites in the rice genome, which needs a single DNA sequence input	Rice genome	Tahir et al., 2019
MM-6mA-Pred	This tool identifies 6mA and non-6mA sites by substantial variations in transition probability among adjacent nucleotides based on Morkov’s model having better prediction compared to i6mA-Pred	Rice genome	Pian et al., 2020
DEEP6mA	Superior performance platform to identify 6mA sites in plants with an overall prediction precision of 94% using a convolutional neural network (CNN) to retrieve high-level sequence features and a bi-directional long-term memory network (BLSTM) to acquire dependence structure along the sequence	Multiple species	Li Z. et al., 2019
FastFeatGen	This predictor uses a machine learning approach with motif features to predict the 6mA sites in the genome. Due to the multi-threading and shared memory mechanism, speed is the advantage of this tool	Multiple species	Khaledur Rahman, 2019
eRice	This prediction tool uses a machine learning approach to predict the 6mA sites in the rice genome	Rice genome	Zhang et al., 2020

A further 6mA benchmark dataset for the rice genome can be denoted as 6mA-rice-Lv (Lv et al., 2019). It contains 154,000 6mA sites-contained sequences as positive samples and the same number of negative samples. They trained and analyzed iDNA6mA-rice on 6mA-rice-Lv dataset by fivefold cross-validation and found that iDNA6mA-rice had strong predictive efficiency. They also trained and tested iDNA6mArice on the 6mA-rice-Chen dataset for the purpose of comparison with i6mA-Pred, and found that iDNA6mArice outperformed i6mA-Pred on the 6mA-rice-Chen dataset (Lv et al., 2019). In another study a high-performance and lightweight, approach called SNNRice6mA was developed to improve the prediction accuracy of DNA 6mA sites in the rice genome (Yu and Dai, 2019). It is based on the architecture of convolutional neural networks. Compared to conventional machine learning approaches, it does not require a manually designed sequence feature and can learn high-level abstract features. On the 6mA-rice-Chen and 6mA-rice-Lv datasets, SNNRice6mA obtained an accuracy of ∼93 and 92%, respectively. In the prediction of DNA 6mA sites in the rice genome, SNNRice6mA performed better than previous methods (Yu and Dai, 2019).

In a subsequent study a 6mA predictor was trained on multi-species data (i.e., *Homo sapiens* (human), *O.* sativa (rice), *Drosophila melanogaster* (fruit fly), and *C. elegans* (worm) from a set of sequence-based features, including position-specific triple nucleotide propensity (PSTNP), physicochemical properties, and electron-ion pseudopotential interaction (EIIP). The key features were selected by performing the maximum relevance maximum distance (MRMD) analysis and the Extreme Gradient Boosting (XGBoost) algorithm was used to construct predictor. The p6mA outperformed using different datasets (Wang et al., 2020). Lv et al. (2020) in a recent study developed a computational approach for identifying 5hmC, 6mA, and 4mC sites in different species. Initially, in order to formulate samples, they used the K-tuple nucleotide portion, mono-nucleotide binary encoding scheme, and nucleotide chemical property and nucleotide frequency. Random forest was subsequently used to recognize 5hmC, 6mA, and 4mC sites. Cross-validated results revealed that in defining the sites of three modification, the proposed approach could generate an outstanding generalization ability. Finally, a predictor called iDNA-MS was developed based on the proposed techniques (Lv et al., 2020). Zhang et al. developed AI based 6mA prediction approach named the eRice database dedicated for providing reliable and efficient genomic and epigenomic resources for both *japonica* and *indica* rice (Zhang et al., 2020). Very recently Hasan et al. developed a machine learning method for predicting 6mA sites in different plant genomes called Meta-i6mA. In an extensive independent test Meta-i6mA outperformed and revealed high Matthews correlation coefficient values of 0.918, 0.827, and 0.635 for Rosaceae, rice and *Arabidopsis thaliana*, respectively (Hasan et al., 2020). Currently, several machine learning approaches have been developed for identifying genome wide 6mA sites, each one has their own limitations. However, there is room for their efficiency to be enhanced to predict 6mA sites in various species.

## Concluding Remarks and Future Perspective

Now, we are known that 6mA has been identified as an important epigenetic mark in both mammals and model plants as the detection approaches have begun to become increasingly sensitive (Liang et al., 2020). However, it will be of importance to study the presence of 6mA across the life cycle from model plants to non-model crops. As horticultural plants often equip with differentiation of cell types or organs and exhibit annual or perennial growth behaviors, using combination of rapidly growing detection approaches, it will be important to identify the biological functions of 6mA and its genome localization patterns in different crops. The outstanding question is whether the biological functions of 6mA in model plants are conserved in plants or it has evolved new biological functions in these organisms.

As 6mA occurs less frequently in more recently evolved mammals and plants, this might reflect a more specialized functional role in crops. Moreover, in model plants, we have known 6mA functions as a complimentary epigenetic mark to 5mC that is in contrast with fungi where the strong negative relation is observed between 6mA and 5mC (Zhang et al., 2018; Liang et al., 2020). In addition, it has been revealed that 6mA is associated with histone modifications in mammals (Greer et al., 2015; Ma et al., 2019). So, it will be of interest to further investigate their interactions and cross-talk signals between 6mA and other types of epigenetic marks in plants and identify weather they combinedly regulate gene activity and function (Figure 1C). With the advancement in detection approaches of 6mA, future studies should focus on the environmental factors or combine with specific developmental cues regulated by 6mA in horticultural plants and its conserved/divergent enzymes at different species level, which will offer clues for its biological significance and evolutionary conservation.

In addition, as deep learning approaches have become a powerful strategy for modeling and prediction “Big Data” in genomics and epigenomics (including 6mA), from our perspective, it will lead to detailed and comprehensive detection of 6mA, its potential functions and localization in diverse plant species, especially for horticultural crops. Thus, detection, identification, and prediction of 6mA in non-model crops using latest sensitive detection methods that developed in model plants will open up a new and exciting chapter of discovery in the field of DNA methylation in plants.

## Author Contributions

SL and CG conceived and designed this review. SC and JL drafted the manuscript and created the figures. PZ and AR helped to reviewing and editing the manuscript. All authors have read and agreed to the published version of the manuscript.

## Conflict of Interest

The authors declare that the research was conducted in the absence of any commercial or financial relationships that could be construed as a potential conflict of interest.
